# Clinical Safety and Efficacy of Ablation for Atrial Fibrillation Patients With a History of Stroke

**DOI:** 10.3389/fcvm.2021.630090

**Published:** 2021-03-10

**Authors:** Zi-liang Song, Shao-hui Wu, Dao-liang Zhang, Wei-feng Jiang, Mu Qin, Xu Liu

**Affiliations:** Department of Cardiology, Shanghai Chest Hospital, Shanghai Jiaotong University, Shanghai, China

**Keywords:** atrial fibrillation, stroke, catheter ablation, safety, efficacy

## Abstract

**Objectives:** To evaluate the clinical safety and efficacy of radiofrequency catheter ablation for atrial fibrillation patients with a history of stroke.

**Methods and Results:** A total of 116 symptomatic, drug-refractory AF patients with a history of stroke, and 1:2 matched patients without a history of stroke were enrolled. Of these, 28 cases occurred stroke within 3 months (Group 1), 88 cases with stroke history longer than 3 months (Group 2), and 232 cases without stroke (Group 3). PVI was performed in all patients, extended to ablation of linear lesions ablation. The periprocedural stroke rates and other procedure-related in-hospital complications did not differ significantly among the three groups. The maintenance rate of SR after the procedure showed no significant difference (*p* = 0.333), 52.7, 66.4, and 70.7% in Group 1, 2, and 3, respectively. Furthermore, the comparison between a history of stroke and those without it were also shown no significant difference (*p* = 0.351).

**Conclusions:** Radiofrequency ablation for AF patients occurred stroke, even within 3 months is safe and effective, without higher periprocedural complication rate and recurrence rate.

## Introduction

Atrial fibrillation (AF) is a clinically common arrhythmia and increases the risk of cardiogenic stroke up to 5-fold (1, 2). Atrial fibrillation patients with a history of stroke maintain a higher risk of stroke up to 21%, combined with an elevated risk of death and disability (3). Stroke can also induce AF through neurogenic mechanisms (4). Current studies found that early control of heart rhythm in patients with atrial fibrillation can reduce the incidence of cardiovascular and cerebrovascular events (5). At present, catheter ablation is considered to be an effective method for the treatment of drug-resistant symptomatic AF. However, few studies have evaluated the clinical efficacy and prognosis of atrial fibrillation ablation with a history of stroke (6, 7). A 5-year retrospective study showed that the incidence of recurrent stroke after ablation was lower than that without ablation (7). However, current guidelines lack recommendations on the timing of radiofrequency ablation in patients with a history of stroke. Considering the possibility of perioperative complications, most centers only consider radiofrequency ablation at least 3 months after stroke. We conducted the current study to evaluate the effect of radiofrequency catheter ablation in patients with atrial fibrillation with a history of stroke. We also investigated the incidence of procedure-related complications in patients with a history of stroke.

## Methods

### Study Population

One hundred and sixteen AF patients with a history of stroke who underwent radiofrequency catheter ablation for the first time in Shanghai Chest Hospital from January 2017 to July 2018 were enrolled. Patients without a history of stroke who underwent radiofrequency ablation during the same period were 1:2 matched. The exclusion criteria are as follows, left atrial appendage thrombosis, active cerebral hemorrhage, severe sequelae of cerebral infarction, or with other conditions that cannot tolerate radiofrequency ablation.

### Electrophysiological Study and Ablation Protocol

All patients underwent circumferential pulmonary vein isolation, additional ablation of linear lesions was performed in persistent AF. External electric cardioversion will be used to achieve termination to sinus rhythm, finally. Details of the ablation protocol were described in our previous studies (8, 9). Briefly, catheter ablation was performed under the guidance of an electro-anatomical mapping system (CARTO, Biosense Webster, CA, USA). Radiofrequency power output was set up as 40 W, 43°C. The duration of each lesion was 20–30 s, and the perfusion rate of saline was 20–25 mL/min. Heparin was administered in all patients, during the ablation procedure, keeping ACT of 300–350 s.

### Complication Assessment

Severe in-hospital complications were defined as cerebrovascular stroke, transient ischemic attack (TIA), cardiopulmonary resuscitation, acute myocardial infarction, emergency cardiothoracic surgery, AV-block 3rd degree, pericardial tamponade, atrioesophageal fistula, phrenic nerve palsy, and major bleeding with intervention. Major adverse cardiac and cerebrovascular events (MACCE) were defined as cerebrovascular stroke, acute myocardial infarction, and death.

### Follow-Up

After ablation, all patients were treated with antiarrhythmic drugs for 2–3 months. ECG and 24-h Holter monitoring were performed at 3, 6, and 12 months, respectively, and then every 6 months. The successful outcome during the follow-up period was defined as the absence of atrial fibrillation or other persistent atrial arrhythmias, after a 3-month blanking period.

### Statistical Analysis

Continuous variables are expressed as mean ± standard deviation, and compared with independent sample *t*-test or non-parametric test, while discrete variables are expressed as percentages and compared with *x*^2^-test. Fisher's exact test was used to compare categorical variables. Kaplan-Meier analysis was used to compare the results between groups. Multivariate Cox regression analysis was performed to determine the significant risk of recurrence of atrial fibrillation and atrial tachyarrhythmia. All significance tests are two-sided, and *P* < 0.05 is considered significant. All statistical analyses were performed using SPSS (IBM, Armonk, NY) version 18.0 and GraphPad software (GraphPad software Inc., La Jolla, CA).

## Results

### Baseline Patient Characteristics

The baseline characteristics with comparison between groups are displayed in Table 1. No significant differences were found between the groups in sex, prevalence of paroxysmal AF, left ventricular end diastolic diameter (LVEDD), frequency of coronary artery disease (CAD), diabetes mellitus (DM), heart failure. The mean age was significantly older in AF with history of stroke than that without stroke (69.9 ± 6.7 vs. 63.7 ± 9.2 years, *p* < 0.01). In addition, the rate of hypertension (75 vs. 49.6%, *P* < 0.05), CHA2DS2 -VASc Score (5.1 ± 1.3 vs. 1.8 ± 1.4, *P* < 0.05) and left atrium diameter (44.8 ± 4.1 vs. 42.0 ± 5.0 mm, *P* < 0.05) in Group 1 were higher than those in Group 3.

**Table 1:** Clinical characteristics of the groups.

	**Group 1 (*n* = 28)**	**Group 2 (*n* = 88)**	**Group 3 (*n* = 232)**	***P*-value**
Age, years	70.9 ± 7.8	69.5 ± 6.4	63.7 ± 9.2	<0.001^*^^†^
Male, *n* (%)	16 (57.1)	62 (72.1)	151 (65.1)	0.479
Paroxysmal AF, *n* (%)	6 (21.4%)	19 (22.1)	63 (27.2)	0.186
Hypertension, *n* (%)	21 (75.0)	48 (54.5)	115 (49.6)	0.022
CAD, *n* (%)	6 (21.4)	15 (17.0)	35 (15.1)	0.220
Diabetes, *n* (%)	7 (25.0)	14 (15.9)	39 (16.8)	0.272
Heart failure, *n* (%)	5 (17.9)	12 (13.6)	27 (11.6)	0.200
CHA_2_ DS_2_-VASc score	5.1 ± 1.3	4.5 ± 1.3	1.8 ± 1.4	<0.001^*^^†^
LAD, mm	44.8 ± 4.1	42.9 ± 5.2	42.0 ± 5.0	0.015^*^
LVEDD, mm	47.0 ± 4.7	48.6 ± 4.5	47.6 ± 4.1	0.096
LVEF, %	60.7 ± 5.4	61.8 ± 5.5	62.7 ± 4.4	0.051^*^

* *p < 0.05, Group 1 vs. Group 3*.

† *p < 0.05, Group 2 vs. Group 3*.

### Procedure-Related Data

Details of the procedures for different study populations are shown in Table 2. All patients successfully underwent pulmonary vein isolation. There was no significant difference in the procedure parameters between the groups (*P* > 0.05), including PVI, linear ablation, SVC isolation, cardioversion, procedure time, ablation time, fluoroscopic time.

**Table 2:** Procedural characteristics of the groups.

	**Group 1 (*n* = 28)**	**Group 2 (*n* = 88)**	**Group 3 (*n* = 232)**	***P*-value**
PVI, *n* (%)	28 (100.0)	88 (100.0)	232 (100.0)	1.00
Linear ablation, *n* (%)	19 (67.9)	61 (69.3)	159 (31.5)	0.532
SVC isolation, *n* (%)	3 (10.7)	6 (6.8)	19 (8.2)	0.496
Cardioversion, *n* (%)	11 (39.3)	31 (35.2)	76 (32.8)	0.66
Procedure time, min	166.0 ± 23.0	158.8 ± 28.0	162.3 ± 42.8	0.635
Ablation time, min	70.9 ± 9.8	74.0 ± 20.4	73.5 ± 8.3	0.509
Fluoroscopic time, min	6.0 ± 1.4	6.4 ± 1.6	6.4 ± 1.0	0.194

### Procedure-Related Complications

There was no significant difference in the overall incidence of complications, and no severe complications was found among the groups (Table 3). No major adverse cerebrovascular and cardiac events occurred in the groups. Inguinal hematomas were present in one case of group 1, one case of group 2, four cases of group 3. Three patients developed pseudoaneurysm and one patient developed arteriovenous fistula, which healed after mechanical compression. In group 2, one case occurred pericardial tamponade and recovered after pericardial drainage. One case in group 2 developed mild phrenic nerve palsy and recovered 1 week after the procedure. Transient ischemic attack was encountered in one patient in group 3 and recovered without any sequelae after treatment. There were no cases of severe pulmonary vein stenosis, atrioesophageal fistula, hemorrhagic events requiring blood transfusion.

**Table 3:** Procedural complications of different groups.

	**Group 1 (*n* = 28)**	**Group 2 (*n* = 88)**	**Group 3 (*n* = 232)**	***P*-value**
MACCE, *n* (%)	0 (0.0)	0 (0.0)	0 (0.0)	1.00
Myocardial infarction, *n* (%)	0 (0.0)	0 (0.0)	0 (0.0)	1.00
Transient ischemic attack, *n* (%)	0 (0.0)	0 (0.0)	1 (0.4)	0.667
Stroke, *n* (%)	0 (0.0)	0 (0.0)	0 (0.0)	1.00
Major hemorrhage, *n* (%)	0 (0.0)	0 (0.0)	0 (0.0)	1.00
Pericardial tamponade, *n* (%)	0 (0.0)	1 (1.1)	0 (0.0)	0.333
Emergency cardiothoracic surgery, *n* (%)	0 (0.0)	0 (0.0)	0 (0.0)	1.00
Resuscitation, *n* (%)	0 (0.0)	0 (0.0)	0 (0.0)	1.00
AV-block III, *n* (%)	0 (0.0)	0 (0.0)	0 (0.0)	1.00
Atrioesophageal fistula, *n* (%)	0 (0.0)	0 (0.0)	0 (0.0)	1.00
Phrenic nerve palsy	0 (0.0)	0 (0.0)	1 (0.4)	0.667
Inguinal hematoma, *n* (%)	1 (3.6)	1 (1.1)	4 (1.7)	0.460
Pseudoaneurysm, *n* (%)	0 (0.0)	1 (1.1)	2 (0.9)	0.634
Arteriovenous fistula, *n* (%)	0 (0.0)	0 (0.0)	1 (0.4)	0.667
Severe complications, *n* (%)	0 (0.0)	1 (1.1)	2 (0.9)	0.634
Overall complications, *n* (%)	1 (3.6)	2 (2.3)	6 (2.6)	0.519

*MACCE = death, acute myocardial infarction, stroke*.

### Follow-Up Outcomes

**Figure 1:**
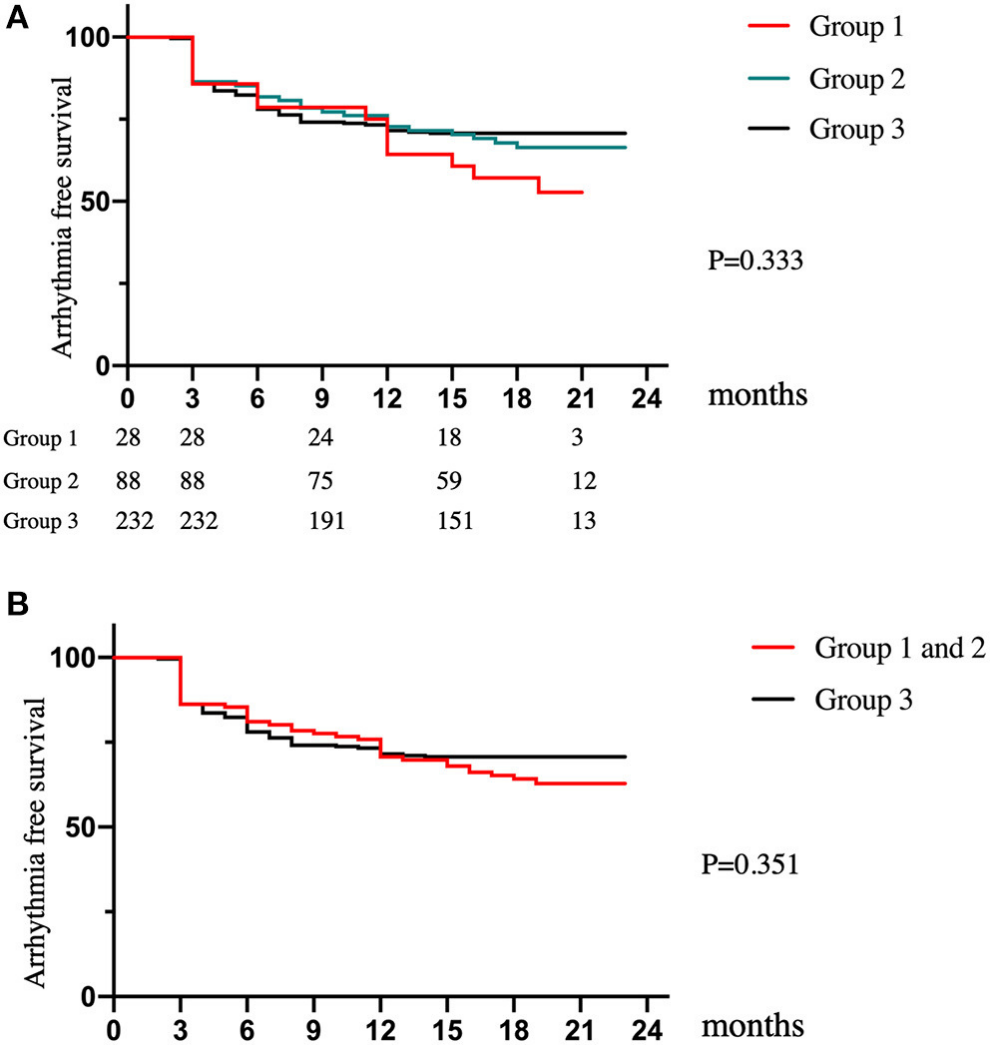
Long-term clinical outcomes. **(A)** The Kaplan–Meier plot of AF/AT-free survival for patients among the three groups. **(B)** The Kaplan–Meier plot of AF/AT-free survival for patients between the patients with a history of stroke (group 1 and 2) and those without it (group 3).

Followed up for 538.8 ± 61.5 days, the SR maintenance rates after the single ablation procedure were 52.7, 66.4, and 70.7% in Groups 1, 2, and 3, respectively. The recurrence-free rates of any atrial tachyarrhythmias and AF at 12 months were 71.4, 76.1, and 78.0% in Groups 1, 2, and 3, respectively. There was no significant difference in recurrence-free survival among the three groups (*p* = 0.333) (Figure 1A). Furthermore, the comparison between the patients with a history of stroke and those without it were also shown no significant difference by log rank test (*p* = 0.351, Figure 1B). Multivariate Cox regression analysis showed no significant difference in AF/AT recurrence between AF patients with and without a history of stroke (HR 1.023, 95% CI 0.562–1.862, *p* = 0.941, Table 4). LA diameter was an independent risk factor for recurrence (*p* = 0.02, Table 4). These results suggest that a history of stroke is not associated with recurrence of AF and any atrial tachyarrhythmias after ablation.

**Table 4:** Multivariate Cox regression analysis for recurrence of AF.

**Variable**	**HR**	**95% CI**	***p*-value**
History of stroke	1.023	0.562–1.862	0.941
LA diameter (mm)	1.045	1.007–1.095	0.020
AF duration	1.001	0.998–1.005	0.393
CHA2DS2-VASc score	1.039	0.869–1.242	0.674
LVEF (%)	1.023	0.975–1.074	0.351

*HR, hazard ratio; CI, confidence interval; LVEF, left ventricular ejection fraction*.

## Discussion

The major findings of our retrospective study: radiofrequency ablation is safe in AF patients who occurred stroke, even within 3 months, and does not increase the incidence of severe cardiovascular and cerebrovascular accidents. In addition, there was no significant difference in SR maintenance rate between AF patients with and without a history of stroke during the follow-up period.

Current clinical guidelines recommend that patients with a TIA or a small stroke initiate or continue anticoagulation early (immediately) (10). Risk of symptomatic intracranial hemorrhage will not increase in AF patients receiving anticoagulants 4–14 days after the acute event, otherwise the recurrence rate of ischemic stroke after mild/moderate stroke will increase significantly with the prolongation of NOAC treatment (11, 12). In addition, patients with AF who survive after stroke can benefit from long-term OAC (13, 14). The patients occurred stroke within 3 months included in our study have been excluded from large stroke and cerebral hemorrhage through preoperative brain imaging examination. These patients had already taken oral anticoagulants before radiofrequency ablation. We conduct perioperative anticoagulation treatments for these patients and strictly control bleeding risk factors (such as uncontrolled hypertension). Our findings confirmed that anticoagulation of patients with stroke during the perioperative period will not increase the risk of cerebral hemorrhage.

The risk of perioperative stroke after catheter ablation for atrial fibrillation ranged from 0.9 to 1.4% (15, 16). Studies about whether the history of stroke increases the risk of stroke after radiofrequency ablation is controversial (4, 16). This may be due to the difference between the application of saline irrigation catheters and improper adherence to anticoagulation approach. Our research results showed that the there was no significant difference in perioperative cardiovascular complications between patients who have a stroke and the control groups. The results of this study are consistent with previous results (6), benefit from periprocedural use of DOACs in AF ablation.

Pericardial tamponade is also one of the main complications of atrial fibrillation ablation, with an incidence ranging from 0 to 6% (17). In this study, a history of stroke did not increase the complications of pericardial tamponade. The incidence of vascular complications during atrial fibrillation ablation varies from 0.2 to 1.5% (18). Uninterrupted anticoagulation therapy will not increase vascular complications (19). There were no significant difference in vascular complications of the three groups of patients.

Prior history of stroke is a significant risk factor for recurrent stroke in AF patients. In addition, the 5-year follow-up results showed that the recurrence rates of stroke, death and heart failure admission in patients receiving ablation was lower compared to those in patients without ablation (7). This frequent follow-up leads to more use of anticoagulants that may significantly reduce the risk of stroke recurrence. Ablation therapy is necessary for AF patients with a history of stroke. Our current study confirmed that the long-term SR maintenance rate of patients with and without a history of stroke enrolled was comparable. No significant difference in the recurrence of atrial tachyarrhythmia following long-term follow-up was confirmed even in patients occurred stroke within 3 months before ablation. Stroke history is not a predictor of AF recurrence after ablation, which is consistent with previous studies (6). Therefore, radiofrequency ablation should also be recommended for patients with stroke history of atrial fibrillation.

## Conclusion

Radiofrequency ablation is safe and effective in the treatment of AF patients occurred mild/moderate stroke even within 3 months. The SR maintenance rate and the incidence of complications are comparable to those in the control group.

## Limitations

This is a retrospective analysis conducted in a single center, and enrolled patients may be biased. The conclusions of this study need to be verified by more patients through multi-center prospective clinical studies. We do not routinely perform MRI to detect cerebral infarction, so the occurrence of silent cerebral ischemia may be underestimated. In addition, although ECG and Holter examinations were performed at each follow-up, we cannot avoid the possibility of recurrence of asymptomatic atrial fibrillation.

## Data Availability Statement

The original contributions presented in the study are included in the article/supplementary material, further inquiries can be directed to the corresponding authors.

## Ethics Statement

The studies involving human participants were reviewed and approved by Institutional Review Board of Shanghai Chest Hospital. The patients/participants provided their written informed consent to participate in this study.

## Author Contributions

All authors listed have made a substantial, direct and intellectual contribution to the work, and approved it for publication.

## Conflict of Interest

The authors declare that the research was conducted in the absence of any commercial or financial relationships that could be construed as a potential conflict of interest.
